# 

**Published:** 1887-09-10

**Authors:** 


### The Hospital.

**An Institution, Family, and Parochial Journal of Hospitals, Asylums, and all Agencies for the Care of the Sick, Criticism and News.**

SATURDAY, SEPTEMBER 10TH, 1887.

### NOTICE TO ADVERTISERS.

THE scale of charges for small prepaid Advertisements—Situations Wanted, Situations Vacant, etc., etc., is as follows :—

| | s. | d. |
|---|---|---|
| One insertion of three lines | 1 | 0 |
| Three „ „ „ | 2 | 6 |
| Per line afterwards | 0 | 6 |

*A line averages eight words.*

All copy for Advertisements must be sent to A. P. WATT, 2, PATERNOSTER SQUARE, E.C., by first post on the Tuesday *preceding* publication.

### NOTICE TO CORRESPONDENTS.

All correspondence must be written on one side of the paper only. We cannot undertake to return MSS. not used.

**Communications,** whether intended for insertion or for private information, must be authenticated by the names and addresses of the writers, not necessarily for publication.

**Local papers** containing reports or news paragraphs **should be marked.**

It is especially requested that early intelligence of **local events of interest,** or which it may be thought desirable to bring under notice, may be sent direct to the Editor.

Communications respecting editorial matters should be addressed to the Editor, Norfolk House, Norfolk Street, Strand, London, W.C.

### THE LITERARY PRIZE.

A PRIZE of **Two Guineas** will be given every month for the best story or incident based upon hospital, or asylum, or student life, or any incident connected with the professions of medicine or nursing.

*** THE CHILDREN'S CORNER and AMUSEMENTS WITH PROFIT will be found on page vi of this week's issue.

### THE IN- AND OUT-PATIENTS' HOSPITAL DIARY.

This Diary is so arranged as to make some portion of it appear every week, and the whole in each monthly part. It has been very difficult to compile, and corrections will at all times be gratefully received. It has been suggested that similar information about the larger Provincial and Scotch Hospitals would be useful to many readers. We should be glad to hear if this is felt to be the case.

### I.—GENERAL HOSPITALS.

| Hospitals and Terms of Admission. | Physicians, Surgeons, and Medical Officers, with Days and Hours of Attendance. |
|---|---|
| Charing Cross Hospital, West Strand, W.C. Sec., Mr. A. E. Reade. Urgent cases are immediately admitted as in-patients. Subscriber's letter expected in other cases. Out-patients admitted without subscriber's letter first time; for continued attendance they must be obtained from the Secretary | *In-Physicians*, Drs. Pollock, M. Th.; Green, W. S.; Bruce, Tu. F. Hour, 1.30<br>*In-Surgeons*, Messrs. Barwell, M. Th.; Bellamy, Tu. F.; Bloxam, W. S. Hour, 2<br>*Out-Physicians*, Drs. Wilcocks, M.Th.; Abercrombie, Tu. F.; Lubbock, W. S.; Murray, W. S. Hour, 1.30<br>*Out-Surgeons*, Messrs. Cantlie, M.Th.; J. H. Morgan, Tu. F.; Stanley Boyd, W. S. Hour, 1.30 |
| "Dreadnought" Hospital (Seamen's Hospital Society), Greenwich, S.E. Sec., Mr. W. T. Evans. Entirely free to seamen and to all accident cases | *In- and Out-Physicians*, Drs. Curnow, Tu. Th. S.; H. T. Griffiths, M.W.F. Daily, between 9 and 3<br>*In- and Out-Surgeons*, Messrs. W. J. Smith, G. R. Turner. Daily, between 9 and 3 |
| French Hospital, 10, Leicester Place, Leicester Sq., W.C. Sec., Mr. E. Rimmel. Free to urgent cases and to all foreigners speaking French | *In-Physician*, Dr. Vintras, Tu. S. 10<br>*In-Surgeons*, Sir W. MacCormac, Tu. 9; Mr. J. Késer, W. F. 10<br>*Out-Physician*, Dr. Vintras, Tu. S. 10<br>*Out-Surgeons*, Messrs. H. de Méric, M. Th. 10; J. Késer, W. F. 10 |
| German Hospital, 113, Dalston Lane, E. Sec., Mr. C. Feldmann. Free to natives of Germany, and others speaking German, and to all accidents and emergencies. Others by Governors' letters<br>——— Eastern Branch, 49, Mansell Street, Aldgate, E.<br>——— Western Branch, 259, Oxford Street, W. | *In-Physicians*, Drs. Weber, M. T. 2; Port, Tu. F. 9 a.m.<br>*In-Surgeons*, Drs. Lichtenberg and Burger, W. S. 2<br>*Out-Physicians*, Drs. Tuchmann, Tu. W. 2; Ludwig, M. Th. 2; Keller, Tu. W. F. 2; Philippi, M. Th. F. 2 (*Men*, Tu. Th.; *Women*, M. W. F.)<br>*Out-Physician*, Dr. C. Harrer, Tu. F. 8 to 10 a.m.<br>*Out-Physician*, Dr. M. Castaneda, M. T. 8 to 10 a.m. |
| Great Northern Central Hospital, Caledonian Road, N. Sec., Mr. W. T. Grant. Free to all, without letters of recommendation | *In-Physicians*, Drs. Cholmeley, Cook, and Burnett, M. Tu. W. Th. F. 2<br>*In-Surgeons*, Messrs. W. Adams, W. Spencer Watson, and J. Macready, M. Tu. W. Th. F. 2<br>*Out-Physicians*, Drs. Beale, M. Th. 2; Beevor, Tu. 2, S. 10<br>*Out-Surgeons*, Messrs. Lockwood, M. Th. 2; Makins, Tu. F. 2 |
| Guy's Hospital, St. Thomas's Street, S.E. Supt., Dr. C. J. Steele. Patients are admitted, if beds are available, without letters of recommendation, on application at the Hospital. Out-patients are seen free without letters of recommendation | *In-Physicians*, Drs. Pavy, Tu. F.; Pye-Smith, M. Th. S.; Taylor, M. Tu. Th. F. Hour, 1.30<br>*In-Surgeons*, Messrs. Bryant, M. Th. Durham, M. Th. F.; Howse, W. S.; D. Colley, M. Th. Hour, 1.30<br>*Out-Physicians*, Drs. Carrington, W.; Hale White, M.; Goodhart, M. Tu. Th. F. Hour, 12.30<br>*Out-Surgeons*, Messrs. Lucas, Th.; Golding Bird, S.; Jacobson, W.; Symonds, M. Hour, 12.30 |
| King's College Hospital, Portugal Street, W.C. Sec., Mr. T. Mosse Macdonald. Accidents and urgent cases free at all times. Other cases by Governors' letters, but where not obtainable, application may be made to the Secretary | *In-Physicians*, Drs. Beale, Duffin, Yeo, Tu. W. F. 1.30; Tu. W. F. S. 2<br>*In-Surgeons*, Mr. Wood, Sir J. Lister, Messrs. H. Smith, W. Rose. Daily, 1.30<br>*Out-Physicians*, Drs. Ferrier, J. Curnow, N. I. C. Tirard. Daily, 1.<br>*Out-Surgeons*, Messrs. W. Rose, W. Cheyne, A. B. Barrow. Daily, 1. |
| London Hospital, Whitechapel Road, E. Sec., Mr. A. Haggard. Accidents and urgent cases free; out-patients for dental and maternity departments need no recommendation; other cases by subscriber's order. Out-patients are required to attend only once, unless otherwise directed by the Physician or Surgeon | *In-Physicians*, Drs. Mackenzie, Tu. F.; Down, Tu. F.; H. Jackson, M. Th.; Sutton, M. Th.; Fenwick, Tu. F. 1.30<br>*In-Surgeons*, Messrs. Rivington, Tu. F.; Couper, Waren Tay, M. Th.; McCarthy, W.S.; Treves, Tu.F. Hour, 1.30<br>*Out-Physicians*, Drs. Sansom, Th.; M. Ralfe, M. Th.; Turner, Tu. F.; Warner, Tu. F.; Gilbart Smith, W. S. J. Anderson, W. S. Hour, 1.30<br>*Out-Surgeons*, Messrs. Mansell-Moullin, M. Th.; Eve, M. W.; Reeves, Tu. S.; H. Fenwick, Tu.F. Hour, 1.30 |

| Hospitals and Terms of Admission. | Physicians, Surgeons, and Medical Officers, with Days and Hours of Attendance. |
|---|---|
| London Temperance Hospital, Hampstead Road, N.W. For treatment without alcohol. By letter or small payment | *In- and Out-Physicians*, Drs. J. Edmunds, M.; J. J. Ridge, Tu. F. Hour, 1.30<br>*In- and Out-Surgeons* Mr. A. P. Gould Th. 2.30 |
| Metropolitan Free Hospital, Kingsland Road, N. Sec., Mr. G. Croxton. Admission free to the sick poor, without any letter of recommendation. Urgent cases at all times | *In- and Out-Physicians*, Drs. Drysdale, Tu. F. 12; J. G. Dudley, W. S. 12; Fotherby, M. Th. 12; H. H. Tooth, Tu. F. 9; A. Haig, W. S. 9; A. Davies, M. Th. 9<br>*In-and Out-Surgeons*, Messrs. E. J. Chance, W. S. 9; Goodsall, Tu. F. 9; Walsham, M. Th. 9; A. A. Bowlby, F. 9; S. Paget, Th. 9; C. J. Thompson, daily, 9 |
| Middlesex Hospital, Berners Street, W. Sec. and Supt., Mr. A. O. D. Bartholeyns. Urgent cases admitted at all times; other cases by ticket. All medical cases must, in the first instance, be seen by the Resident Medical Officer, who attends at the hospital daily, at 11 a.m. | *In-Physicians*, Drs. Cayley, M. Th.; Coupland, Tu. F.; D. Powell, M.Th.; Finlay, Tu. F. Hour, 1.30<br>*In-Surgeons*, Messrs. Hulke, M. Th.; Lawson, M. Th.; Morris, Tu. F. Hour, 1.30<br>*Out-Physicians*, Drs. Fowler, Tu. F. 3.30; Biss, M. Th. 1.30; Pringle, W. S. 1.30<br>*Out-Surgeons*, Messrs. Andrew Clark, M. 1.15 (*Men*); Th. 1.15 (*Women*); Pearce Gould, F. 1.30 (*Men*); Tu. 1.30 (*Women*); J. B. Sutton, W. S. 9 |
| Miller Hospital and Kent Dispensary, Greenwich, S.E. Hon. Sec. Mr. W. Bristow. | *Physicians*, Drs. T. Creed, C. H. Hartt, G. Willes<br>*Surgeons*, Messrs. T. Moore, J. Poland, E. Clarke |
| North-West London Hospital, 18, Kentish Town Road, N.W. Sec., Mr. A. Craske. By subscribers' tickets or payment, but accidents and urgent cases free at all times | *In- and Out-Physicians*, Drs. W. C. Hood, J. Shaw, Edwarde H. Campbell. Daily, 1.30<br>*In- and Out-Surgeons*, Messrs. F. Durham, Collier, Jennings, J. Black. Daily, 1.30 |
| Ospedale Italiano, 41, Queen Square, Bloomsbury. Sec., Sig. L. Bonacina. Free to all, but Italians have preference | *In- and Out-Physicians*, Drs. M. Castaneda, Tu. F. 10 to 11; B. Sassella, M.Th. 10 to 11; Mr. J.W.B. Steggall, W.S. 4 to 5 |
| Poplar Hospital, 303, East India Dock Road, E. Sec., Lt.-Col. Feneran. Free to accidents and emergencies | *In-Surgeons*, Messrs. F. M. Corner, M. Brownfield, and Resident Surgeons<br>*Out-Surgeons*, Dr. J. D. Grant, W. S. Mr. T. E. Bowkett, Tu. F. 12 to 2 |
| Royal Free Hospital, Gray's Inn Road, W.C. Sec., Mr. J. S. Blyth. Admission free to the sick poor, without any letter of recommendation | *In- and Out-Physicians*, Drs. Cockle, M. Th.; Sainsbury, Tu. F.; West, W. S. Hour, 1<br>*In- and Out-Surgeons*, Messrs. Rose, M. Th.; W. Gant, Tu. F.; Barrow, W. S. Hour, 1 |
| St. Bartholomew's Hospital, Smithfield. Clerk, Mr. W. Cross. Admission free, without letter or ticket. Accidents and other urgent cases admitted at any time; ordinary cases of disease daily, between 9 and 10. Patients are seen for Electrical treatment on M. Tu. Th. and F. 1.30 | *In-Physicians*, Drs. Andrew, Church, Gee, Sir D. Duckworth. Daily, 1.30<br>*In-Surgeons*, Messrs. Savory, T. Smith, Willett, Langton, M. Baker. Daily, 1.30<br>*Out-Physicians*, Drs. Hensley, M. Th. 11; Brunton, Tu. F. 11; Legg, W. S. 11; N. Moore, Tu. W. Th. S. 9<br>*Out-Surgeons*, Messrs. Marsh, Tu. F.; 12; Butlin, M. Th. 12; Walsham, W. S. 12; Cripps and Bruce Clarke, daily, 9<br>*Casualty Physicians*, Drs. Davies, Tu. W. F. S.; O. Browne, M. Tu. Th. F.; Nias, M. W. Th. S. Hour, 9 |
| St. George's Hospital, Hyde Park Corner, S.W. Sec., Mr. C. L. Todd. Free to accidents and emergencies. Other in-cases by Governor's or subscriber's letter, but, in necessitous cases, this recommendation is not strictly insisted on. Letters are not required for out-patients. Vaccination on Th. at 11 | *In-Physicians*, Drs. Wadham, M. F.; Dickinson, M. Tu. Th. F.; Whipham, Tu.W. S.; Cavafy, Tu. S. Hour, 1<br>*In-Surgeons*, Messrs. Holmes, M. Th. F. 1, W. 1.30; Rouse, M. Th. F. 1, W. 1.30; Haward, Tu. Th. S. 1, W. 1.30<br>*Out-Physicians*, Drs. Ewart, M. F.; Owen, Tu. S. Hours, 10.30 to 12<br>*Out-Surgeons*, Messrs. Bennett, M. F.; Dent, Tu. S. Hours, 10.30 to 12 *Men*, F. S.; *Women*, M. Tu. |

### THE CHILDREN'S CORNER.

**Fourth Quarterly Prize Competition.**
Began 2nd July, 1887; ends 24th Sept., 1887.

**Puzzles—Eleventh Week.**

No. 42. EPITAPH PUZZLE.

Bene A. Thin Thed Ustt H. E. M. O. Uld yo

L. D. C. RUSTO! Fnel L. B.

Ach EL. Lor. Lat. Ely.

Wa S. shove N. W. How-Ass! kill'd I. N. T. H.

Ear T Sofp I Es-cu Star

D. San D. T Art. San D. K. N. E. W. E.

Ver-Yus. E.—Oft He ove N. W. Hens He

'Dli V'DL on geno Ug H. S. hem A. D. E. he R.

La Stp U f-f. No Wheres He dot H. L. i.e.

in hop Esthathe R. C. R U S. T W. I.

L L B Era is 'D——!

No. 43. CHARADE.

My *first* we live on, my *second* is a prophet,
And my *whole* a great painter.

No 44. CONUNDRUM. When is a small fishpond like a birdcage?

No. 45. Cut off my head and singular I appear, cut off my tail and plural I appear; cut off both head and tail, O wondrous fact, nothing remains although my middle's here.

**Letters—Eleventh Week.**

Credit will be given next week for the best "Dog Story."

**Results of Ninth Week—Puzzles.**

No. 34. ORNITHOLOGICAL ENIGMA. The bird which is seldom seen except at night is the *owl*, the one who on the battlefield calls others to the fight, the *trumpeter*. The bird which each word will repeat, the *mocking-bird*; and the one which every day you meet, is of course the *sparrow*.

No. 35. DECAPITATIONS. Forage, orage, rage, age.

No. 36. TRANSPOSITIONS. The towns are as follows:

| | |
|---|---|
| Montauban. | Londonderry. |
| Aberdeen. | Allahabad. |
| Ravenna. | Navarre. |
| Yeovil. | Darmstadt. |

The initials form "Maryland," one of the United States.

No. 37. RIDDLE-ME-REE.—A River.

*All right*—Umslopogaas, The Eda; *Three right*—Skye, Ossian, Scrooge, A. G. S. McCulloch, A. C. K., Toby, Mount Edgcumbe; *Two right*—Princess Ida, Socrates, Poodle, Fern, Ellie.

**Letters.**

The replies to "your favourite way of spending a wet day in the holidays" are not very numerous. Princess Ida describes a game which may be new to many of my readers, and will perhaps afford them amusement to try. She says:

Rather a favourite amusement of ours is what we call "Parliament," though I do not know whether it much resembles its namesake. We have each to prepare a paper on any subject we like, and take it in turns reading them out; then there is a grand debate after the reading of each paper; and really it is great fun. Of course we are each supposed to be some celebrated politician, and each do their best to sustain their character. We have not had a parliamentary debate for some time, but I daresay it will regain favour during the autumn days. We started a "Gazette" about five years ago, each writing articles, poems, etc., for it, and we have kept it up ever since, during the autumn and winter.

The institution of the Gazette must be a source of great amusement during the winter. Toby relates the story of some leather leggings which on a wet afternoon he rummaged from an old trunk. They were the property of a Spanish robber who was discovered by two English officers in the cupboard of a wayside inn, where on a stormy night they had taken shelter. They found the ruffian with two daggers in his possession, one of which was concealed in his leggings. He was quickly disarmed and handed over to justice, the leggings being retained as a trophy.

Skye gives her experience of a wet holiday at the sea-side and at home:

I think a wet holiday at the sea-side or lodging away from home is rather miserable, because one has so few resources. Reading for a little, then stare out at the sitting-room window perhaps to fix your eyes on a brick wall; from this we turn inside once more and begin to draw, soon get drowsy, yawn, and give it up; resolve to go out booted and waterproofed for a walk along the beach; see nobody about but a few grumpy fishermen gazing out to sea; return to our quarters wishing it were bed-time. At home one can defy a wet holiday—I rather like it. I sometimes do quite a large baking, cake, gingerbread, and tea-cakes, etc.; after that we children get up a small tea-party on our own hook, which is conducted with much ceremony, tickets of admission, and full dress indispensable, then cheering and speeches to finish off; then a good romping game. Having all helped to tidy up, we sit down to hear some nice story read aloud. Here endeth a jolly wet holiday "at home." Once we got up an impromptu pantomime, which almost ended tragically. My brother Bob was Bluebeard. Just as he was going to cut my head off (I was Fatima) a lighted candle caught his beard and it flared up. He was not hurt much, for papa threw something round him and put the blaze out in a minute; that was in the Christmas holidays.

Three marks each:—Skye, Princess Ida, Ellie, Toby, Mount Edgcumbe, Socrates, Ossian.

Answers must be addressed to the "PUZZLE EDITOR," THE HOSPITAL, Norfolk House, Norfolk Street, Strand, W.C., not later than Thursday next. The "Children's Corner" Coupon (see advertisement pages) must be enclosed.

The address and full Christian name and surname, and the age of the competitor, must be stated in all replies. A *nom de plume* may be added for the purposes of publication.

### AMUSEMENTS WITH PROFIT.

### PRIZE COMPETITIONS.

**Quarterly Prize Competition.**
Began 23rd July; ends 15th October.

**A Prize of Three Guineas**
will be awarded to the Competitor who shall have sent in the largest number of correct or best answers. No one will be permitted to win the Three Guinea Prize twice in any one year, but we shall award annually an additional
**Prize of Five Guineas**
to the competitor who shall have sent in the largest number of correct or best answers during the twelve months.

**No. 15. Double Acrostic.**

Although my first must finally prevail,
My finals oft my first assail.

This plant, tradition says, uprose
To rescue Scotia from her foes.

Kept keen I deftly do my duty;
Neglect me and I spoil your beauty.

'Twas here, to ease remorse's mood,
'Mid rain great Sam bare-headed stood.

A highly tempered kind of blade,
To our brave soldiers not purvey'd.

'Twas thus they poor Polonius buried,
To death by Hamlet's rapier hurried.

**No. 16. Rhythmical Recreation.**

On "Home."

| | |
|---|---|
| Blessed | blest |
| With | serene |
| More | rest |
| Amid | mein |
| While | one |
| Whose | need |
| Forth | done |
| To | indeed |

**Answers.**

No. 11. DOUBLE ACROSTIC.

| | | |
|---|---|---|
| G | am | E |
| R | oya | L |
| E | l | I |
| E | lipha | Z |
| N | iagar | A |
| W | eb | B |
| I | nnocenc | E |
| C | hea | T |
| H | ealt | H |

Correct answers have been received from Boss, Esau, Gretta, Common Sense, Aralc, Perseverance, Toby, Mater.

No. 12. SYMPATHY.

The definitions of the above subject are not, on the whole as good as usual; the following is one of the best.

Sympathy! best sentiment of human hearts!
Feeling with others, whether in joy or grief;
This bond of fellow-feeling makes us all
As one great family, though of many parts;
To stand beside the couch of hopeless pain,
Soothing with tender care the sufferer's woe,
Is sympathy's best work; freely to give
A due proportion of what means we have
To help our fellows in misfortune cast,
Exemplifies an active sympathy,
To comfort mourning sorrow-laden hearts,
Brings out in fullest force, sweet sympathy.—K. M.

Answers have been received from K. M., Mater, Ellice, Brownie, Perseverance, Condorcet, Gretta, V. W., Aralc, Toby, Man of the Sea, Fred C.

Answers must be addressed to THE PRIZE EDITOR, AMUSEMENTS WITH PROFIT, THE HOSPITAL, Norfolk House, Norfolk Street, Strand, W.C., not later than the *first* post on Friday next. The full name and address must in all cases be given, but a *nom de plume* may be added for publication. Only one side of the paper should be written on. The "Amusements with Profit" Coupon (see advertisement pages) must be enclosed with replies.

